# How can distinct egg polymorphism be maintained in the rufescent prinia (*Prinia rufescens*)–plaintive cuckoo (*Cacomantis merulinus*) interaction—a modeling approach

**DOI:** 10.1002/ece3.3090

**Published:** 2017-06-15

**Authors:** Wei Liang, Canchao Yang, Fugo Takasu

**Affiliations:** ^1^ Ministry of Education Key Laboratory for Tropical Plant and Animal Ecology College of Life Sciences Hainan Normal University Haikou China; ^2^ Department of Information and Computer Science Nara Women's University Kita‐Uoya Nishimachi Nara Japan

**Keywords:** avian brood parasitism, coevolution, egg phenotype, frequency‐dependent selection, population genetics model

## Abstract

In avian brood parasitism, both the host and the parasite are expected to develop various conflicting adaptations; hosts develop a defense against parasitism, such as an ability to recognize and reject parasitic eggs that look unlike their own, while parasites evolve egg mimicry to counter this host defense. Hosts may further evolve to generate various egg phenotypes that are not mimicked by parasites. Difference in egg phenotype critically affects the successful reproduction of hosts and parasites. Recent studies have shown that clear polymorphism in egg phenotype is observed in several host–parasite interactions, which suggests that egg polymorphism may be a more universal phenomenon than previously thought. We examined the mechanism for maintaining egg polymorphism in the rufescent prinia (*Prinia rufescens*) that is parasitized by the plaintive cuckoo (*Cacomantis merulinus*) from a theoretical viewpoint based on a mathematical model. The prinia has four distinct egg phenotypes: immaculate white, immaculate blue, white with spots, and blue with spots. Only two egg phenotypes, white with spots and blue with spots, are found in the cuckoo population. We show that the observed prinia and cuckoo phenotypes cannot be at an equilibrium and that egg polymorphism can be maintained either at stationary equilibrium or with dynamic, frequency oscillations, depending on the mutation rates of the background color and spottiness. Long‐term monitoring of the prinia–cuckoo interaction over a wide geographic range is needed to test the results of the model analyses.

## 1 INTRODUCTION

Avian brood parasites exploit parental care of their hosts at the expense of the host's reproductive success (Davies, 2000; Rothstein, 1990). This parasitic pressure is expected to select for host defenses to reduce the reproductive losses caused by parasitism. Host defenses, in turn, will select for counterdefenses by the parasite that defeats the host defense. Indeed, it has been established that many hosts affected by avian brood parasites have evolved a fine‐tuned ability to recognize and reject parasitic eggs that look unlike their own (Davies & Brooke, 1988; Moksnes et al., 1991; Rothstein, 1975; Soler, 2014). The common cuckoo (*Cuculus canorus*), one of the best‐studied brood parasites, has evolved sophisticated egg mimicry that prevents host recognition and egg rejection (Brooke & Davies, 1988; Davies, 2011; Honza, Moksnes, Røskaft, & Stokke, 2001; Moksnes & Røskaft, 1995).

In response to egg mimicry by the cuckoo, a host species may develop reduced intraclutch and increased interclutch variations in egg phenotype, which would require the cuckoo to mimic a particular egg phenotype in order to successfully parasitize the nest (Øien, Moksnes, & Røskaft, 1995; Stokke, Moksnes, & Røskaft, 2002; Stokke, Takasu, Moksnes, & Røskaft, 2007). Such a coevolutionary arms race might lead to polymorphism in egg phenotype (Tanaka, 2016; Yang, Li, Liang, & Møller, 2016; Yang et al., 2010).

Yang et al. (2010) demonstrated that the ashy‐throated parrotbill (*Paradoxornis alphonisianus*), a host of the common cuckoo in South China, shows clear polymorphism in egg color with three distinct phenotypes (white, pale blue and blue eggs) that also occur in the cuckoo. The vinous‐throated parrotbill (*P. webbianus*) in Korea also shows clear dimorphism with white or blue eggs (Kim, Yamagishi, & Won, 1995). Both parrotbill species are consistently able to recognize and reject a cuckoo egg that looks unlike their own in the clutch (Lee, Kim, & Yoo, 2005; Lee & Yoo, 2004; Yang et al., 2010) and it has been suggested that the egg color polymorphism observed in the parrotbill and the cuckoo has evolved as a result of antagonistic coevolution (Lee & Jabłoński, 2012; Yang et al., 2010).

Liang et al. (2012) studied how polymorphism with three phenotypes can be maintained in the parrotbill–cuckoo interaction using a mathematical modeling approach. They constructed a population genetics model and analyzed how the frequencies of the three egg types change with time. The model analysis suggested that polymorphism is likely maintained dynamically; the frequency of each type oscillates within a certain period and is primarily dependent on the parasitism rate.

Yang, Huang, et al. (2016) demonstrated that the plaintive cuckoo (*Cacomantis merulinus*) and the common tailorbird (*Orthotomus sutorius*) have evolved dimorphic white and blue egg phenotypes with brownish spots. The matching egg appearance between plaintive cuckoos and common tailorbirds was presumably a result of negative frequency‐dependent selection, the same as in the parrotbill–cuckoo interaction (Liang et al., 2012).

**Figure 1:**
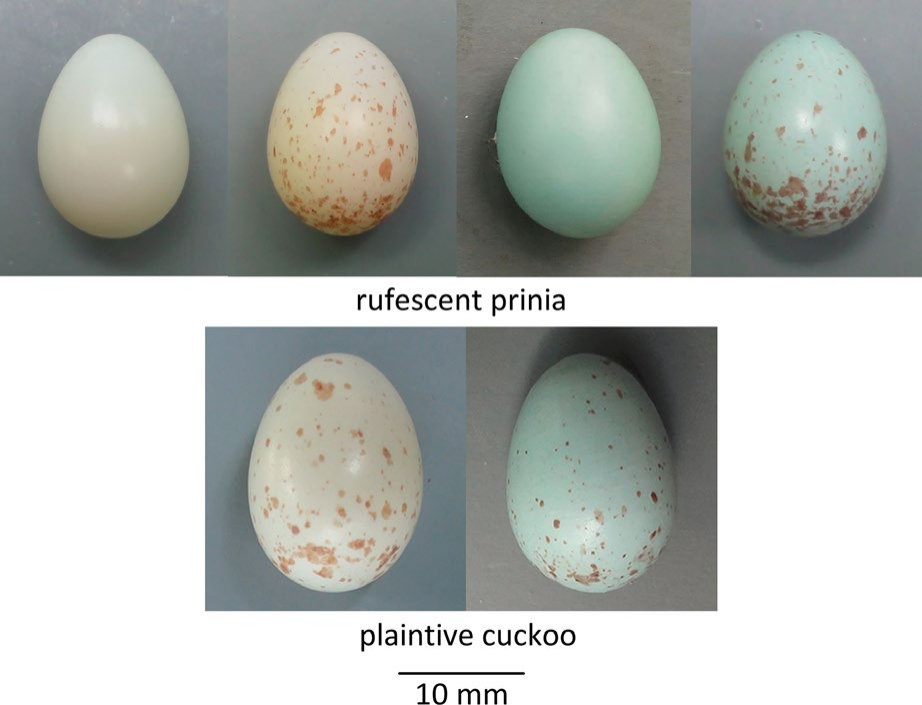
Color photographs of the four egg phenotypes in the rufescent prinia (top) and two in the plaintive cuckoo (bottom). Four distinct phenotypes, immaculate white, white with spots, immaculate blue, and blue with spots, in the prinia and two phenotypes, white with spots and blue with spots, in the cuckoo are clearly shown. Photograph by Longwu Wang

Recently, it has been shown that the rufescent prinia (*Prinia rufescens*), another host of the plaintive cuckoo breeding sympatrically within the same area, has four distinct egg phenotypes: immaculate white and blue eggs without spots, and white and blue eggs with brownish spots. Only one type of eggs is found in a clutch (Yang, C., Wang, L., Zhou, B., Liang, W., Møller, AP, unpubl. data). However, the plaintive cuckoo has only two distinct egg types, either white or blue with brownish spots, that seemingly mimic host eggs (Yang, Huang, et al., 2016). Figure 1 shows these egg types observed in the rufescent prinia and the plaintive cuckoo. Although no quantitative analysis of egg color and spots has yet been done for the rufescent prinia (but see Yang, Huang, et al., 2016 for the plaintive cuckoo and the common tailorbird), distinct polymorphism is obvious.

A question then arises as to how these distinct egg phenotypes can be maintained in the rufescent prinia and the plaintive cuckoo populations. Although the interaction between the rufescent prinia and the plaintive cuckoo is seemingly similar to that of the parrotbill and the common cuckoo, the former could be different from the latter in the expression of egg phenotype; the color (white/blue) and the presence/absence of spots may be controlled by independent genes. Therefore, egg phenotype can be considered a two‐dimensional trait color (white/blue) and spottiness (immaculate/spots), while egg phenotype in the latter case is one‐dimensional with color (white/pale blue/blue) as the only trait involved.

In this study, we aim to explore how egg polymorphism can be maintained in the interaction between the rufescent prinia and the plaintive cuckoo from a theoretical viewpoint. We construct a population genetics model using the same approach as Liang et al. (2012), but with a new assumption considered for mutations of egg phenotypes. Based on the model analysis, we suggest that (1) the observed state of four egg phenotypes in the rufescent prinia and two in the plaintive cuckoo cannot be at equilibrium, and (2) the two egg types we have not yet observed in the plaintive cuckoo (immaculate white and blue) will spread if they appear due to a mutation. We also discuss the apparent absence of the two phenotypes in the plaintive cuckoo population.

## 2 THE MODEL

We assume that there are four distinct egg phenotypes in both the host and the parasite population. Although only two phenotypes (white with spots and blue with spots) have been observed in the plaintive cuckoo in South China (Yang, Huang, et al., 2016), this allows the model to deal with general situations that may occur in a future evolutionary time scale. Detailed genetic mechanisms underlying the inheritance of egg phenotype largely remain unknown. However, it is likely that egg phenotype is maternally inherited by female offspring with no paternal influence on phenotype (Fossøy et al., 2016; Gibbs et al., 2000; Gosler, Barnett, & Reynolds, 2000). We therefore assume that egg phenotype is maternally inherited in the model. We denote each of the four phenotypes as 1 (immaculate white), 2 (immaculate blue), 3 (white with spots), and 4 (blue with spots). Let *h*_*i*_ and *p*_*i*_ be the frequency of phenotype *i* in the host and the parasite population, respectively (*i* = 1, 2, 3, 4).

We assume that a proportion *P* of host nests are parasitized (0 <*P*< 1) and that nests are parasitized randomly, independent of phenotype (Antonov et al., 2012; Yang, Takasu, Liang, & Møller, 2015; Liang, Yang, & Takasu, 2016; Yang, Wang, Liang, & Møller, 2016; Yang, Huang, et al., 2016; but see Cherry, Bennett, & Moskát, 2007; Honza, Sulc, Jelínek, Pozgayová, & Procházka, 2014). Multiple parasitism is ignored as we implicitly assume a low parasitism rate *P* (but see Moskát & Honza, 2002; Takasu & Moskát, 2011). Removal of a host egg by a parasite is also ignored in order to simplify the model.

We assume that all hosts have the same ability to recognize and reject unlike eggs. Let *A*(*i*,* j*) represent the probability that a host with egg type *i* accepts a parasitic egg type *j* laid in the nest. It has been demonstrated that the greater the difference in egg phenotype, the lower the probability of parasite egg acceptance (Higuchi, 1998; Stokke et al., 2007; Takasu, 2003; Yang et al., 2010). We then assume that a host will accept parasite eggs according to the following rules; *A*(*i*,* j*) = *A*_0_ when there is no difference in egg phenotype, *A*_*c*_ when only color differs, *A*_s_ when only spottiness differs, and *A*_*cs*_ when both color and spottiness differ (1 ≥ *A*_0_ ≥ *A*_c_ *, A*_s_ *≥ A*_c _≥ 0). Table 1 summarizes the acceptance probabilities *A*(*i*,* j*) for *i*,* j* = 1, 2, 3, 4.

**Table 1:** Acceptance probabilities, *A* (*i*,* j*), for all combinations of egg type (*i*,* j* = 1, 2, 3, 4). Columns represent host phenotype *i* and rows parasite phenotype *j*. In general, 1 ≥ *A*_0_ ≥ *A*_c_, *A*_s_ ≥ *A*_cs_ ≥ 0

	*i* = 1	*i* = 2	*i* = 3	*i* = 4
*j* = 1	*A*_0_	*A*_c_	*A*_s_	*A*_cs_
*j* = 2	*A*_c_	*A*_0_	*A*_cs_	*A*_s_
*j* = 3	*A*_s_	*A*_cs_	*A*_0_	*A*_c_
*j* = 4	*A*_cs_	*A*_s_	*A*_c_	*A*_0_

We also assume that each phenotype can mutate. Specifically, we assume that both the color and the spottiness mutate reciprocally; white or blue eggs change to blue or white eggs, respectively, with the probability *m*_c_, and immaculate or spotted eggs change to spotted or immaculate eggs, respectively, with the probability *m*_s_. No empirical data are available to estimate these mutation probabilities; however, the values would be very small and likely in the order of 10^−4^ (Bürger, Willensdorfer, & Nowak, 2006). In avian brood parasitism, both the host and the parasite have similar generation times. Thus, we assume that both the host and the parasite share the same mutation probabilities.

Using **h** = (*h*_1_, *h*_2_, *h*_3_, *h*_4_)^T^ and **p** = (*p*_1_, *p*_2_, *p*_3_, *p*_4_)^T^ as column vectors, the phenotype frequencies at the next generation **h’** and **p’** are given as follows:

(1) $${\text{h}}^{\prime }=\frac{1}{{\bar{w}}_{H}}\text{M}\;{\text{W}}_{H}\text{h}$$

(2) $${\text{p}}^{\prime }=\frac{1}{{\bar{w}}_{P}}\text{M}\;{\text{W}}_{P}\text{p}$$

Here, ${\bar{w}}_{H}$ and ${\bar{w}}_{P}$ refer to the average fitness, **W**_H_ and **W**_P_ refer to the respective selection matrices, and **M** is the mutation matrix. See Appendix for the derivation.

In this model, we assume an infinitely large population, random mating, and nonoverlapping generations.

The coupled dynamics, equations (1) and (2), describe temporal changes in the frequencies *h*_*i*_ and *p*_*i*_ (*i* = 1, 2, 3, 4). In the next section, we analyze the frequency dynamics with a special focus on the stability of equilibria where (1) all four egg types coexist in both the host and the parasite populations, and (2) immaculate eggs are absent in the parasite population as found by Yang, Huang, et al., (2016).

## 3 RESULTS

### 3.1 Local stability of equilibrium

At equilibrium of equations (1) and (2) with nonzero mutations (*m*_c_, *m*_s _> 0), frequencies of all types have to be equal (see Appendix). By symmetry of the model, there exists a unique internal equilibrium, h* and p*, where all the four types coexist with equal frequency.

(3) $${\text{h}}^{*}=\left(\frac{1}{4},\frac{1}{4},\frac{1}{4},\frac{1}{4}\right)\text{and}\;\;{\text{p}}^{*}=\left(\frac{1}{4},\frac{1}{4},\frac{1}{4},\frac{1}{4}\right)$$

Local stability of an equilibrium can be checked by the magnitude of eigenvalues of the linearized dynamics around the equilibrium (Murray, 2007). A threshold exists for the mutation probability in color and spottiness, respectively, and the equilibrium (3) is locally stable when both the mutation probabilities are larger than the thresholds. Otherwise, the equilibrium is unstable and phenotype frequencies continue to oscillate with a period dependent on the acceptance probabilities *A*_0_, *A*_c_, *A*_s_, and *A*_cs_, the parasitism rate *P*, and the mutation probabilities *m*_c_ and *m*_s_. When unstable, the oscillation period *T* is proportional to the inverse of the square root of the parasitism rate *P* (Appendix).

**Figure 2:**
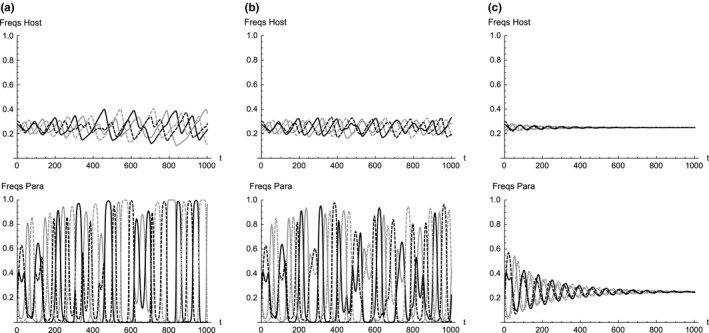
Frequency dynamics of four phenotypes in both the host (top) and the parasite population (bottom) for 1,000 generations. Mutation probabilities are (a) *m*_c_ = *m*_s_ = 1.0 × 10^−10^, (b) *m*_c_ = *m*_s_ = 1.0 × 10^−4^, and (c) *m*_c_ = *m*_s_ = 4.0 × 10^−3^. Initial frequencies are set arbitrarily as (*h*_10_, *h*_20_, *h*_30_, *h*_40_) = (0.225, 0.275, 0.2, 0.3) and (*p*_10_, *p*_20_, *p*_30_, *p*_40_) = (0.275, 0.225, 0.3, 0.2) near the equilibrium (3). Other parameters used are *A*_0_ = 0.8, *A*_c_ = *A*_s_ = 0.1, *A*_cs_ = 0.01, and *p* = .05. For these parameter values, the threshold mutation probabilities are *m*_c_* = *m*_s_* = 1.944 × 10^−3^. Immaculate white, immaculate blue, white with spots and blue with spots is shown in solid gray, solid black, dashed gray and dashed black, respectively

Figure 2 shows typical frequency dynamics when all four types are present in both the host and the parasite populations. As the mutation probabilities are increased from zero beyond the threshold, the equilibrium (3) can be stabilized. For sufficiently small mutation probabilities, the dynamics apparently converge to a heteroclinic cycle (Seger, 1988) in which one phenotype dominates for a longer time but eventually is taken over by another phenotype (Figure 2a). When the mutation probabilities are increased but stay below the thresholds, the dynamics show a sustained but complex oscillation with various frequency modes (Figure 2b). Note that when oscillation occurs, the amplitude is larger in the parasite population than in the host population. This is because all parasite eggs are subjected to the host decision to either reject or to accept, while only a proportion *P* of host nests are under parasitic pressure. When both the mutation probabilities are larger than the thresholds, the dynamics converge to the equilibrium (3) where all four phenotypes coexist stably with equal frequency (Figure 2c).

### 3.2 Do the observed frequencies of egg phenotypes reflect a stable equilibrium?

The rufescent prinia has four egg types but only two types have been found in the plaintive cuckoo (Figure 1); immaculate white and blue eggs have not been observed in the cuckoo population (Yang, Huang, et al., 2016). For this state to be in equilibrium, the probability of a spottiness mutation has to be zero (*m*_s_ = 0) because, otherwise, parasites with immaculate eggs exist because of mutation.

By symmetry of the model, there exists a semi‐internal equilibrium where immaculate eggs are absent in the parasite population.

(4) $$h^{*}=\left(\frac{1}{4},\frac{1}{4},\frac{1}{4},\frac{1}{4}\right)\text{and}\;\;{\text{p}}^{*}=\left(0,0,\frac{1}{2},\frac{1}{2}\right)$$

However, this equilibrium (4) exists only for a special case where the presence or absence of spots does not affect acceptance probabilities at all (*A*_0_ = *A*_s_, *A*_c_ = *A*_cs_). This special case, however, seems not to be applied to the rufescent prinia because the prinia can recognize and reject unlike eggs based on the presence or absence of spots (Yang, C., Wang, L., Zhou, B., Liang, W., Møller, AP, unpubl. data) (*A*_0 _> *A*_s_, *A*_c _> *A*_cs_), and thus, this state (4) cannot be an equilibrium of the dynamics (1) and (2) even when *m*_s_ = 0.

**Figure 3:**
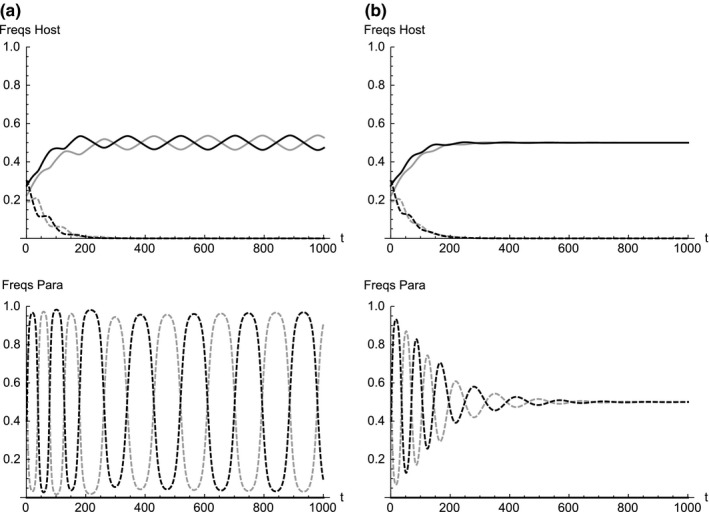
Frequency dynamics in which immaculate white and blue eggs are completely absent in the parasite population (*p*_1_ = *p*_2_ = 0) and no mutation occurs in spottiness *m*_s_ = 0. Frequencies in the host (top) and the parasite (bottom). Probability of mutation in color is (a) *m*_c_ = 1.0 × 10^−4^ and (b) *m*_c_ = 4.0 × 10^−3^. Initial frequencies are set arbitrarily as (*h*_10_, *h*_20_, *h*_30_, *h*_40_) =  (0.225, 0.275, 0.2, 0.3) and (*p*_10_, *p*_20_, *p*_30_, *p*_40_) = (0, 0, 0.3, 0.2). Other parameters used are *A*_0_ = 0.8, *A*_c_ = *A*_s_ = 0.1, *A*_cs_ = 0.01, and *p* = 0.05. Immaculate white, immaculate blue, white with spots and blue with spots is shown in solid gray, solid black, dashed gray and dashed black, respectively

Figure 3 shows typical frequency dynamics when no mutation occurs in spottiness (*m*_s_ = 0), immaculate eggs are completely absent in the parasite population (*p*_1_ = *p*_2_ = 0), and the host can discriminate against the presence or absence of spottiness (*A*_0 _> *A*_s_, *A*_c _> *A*_cs_). In the presence of the parasite eggs with spots, the frequency of host eggs with spots decreases to zero and eventually the host has only immaculate eggs (both *h*_3_ and *h*_4_ converge to zero). When the mutation in color *m*_c_ is small enough, the dynamics converge to an oscillation where hosts with immaculate eggs and parasites with spotted eggs oscillate around an equal frequency of 0.5 (Figure 3a). When the mutation in color is larger than a threshold, the dynamics converge to an equilibrium where the host and parasite each have immaculate white and blue eggs with an equal frequency of 0.5 (Figure 3b). Eventual extinction of hosts with spotted eggs in such a situation occurs irrespective of the mutation probability in color *m*_c_. This is because hosts with immaculate white or blue eggs always have an advantage over hosts with white or blue eggs with spots. Therefore, the observed state of four egg types in the prinia and two egg types in the cuckoo cannot be maintained at equilibrium even if no spottiness mutations occur.

### 3.3 Can immaculate white and blue eggs spread in the parasite population?

**Figure 4:**
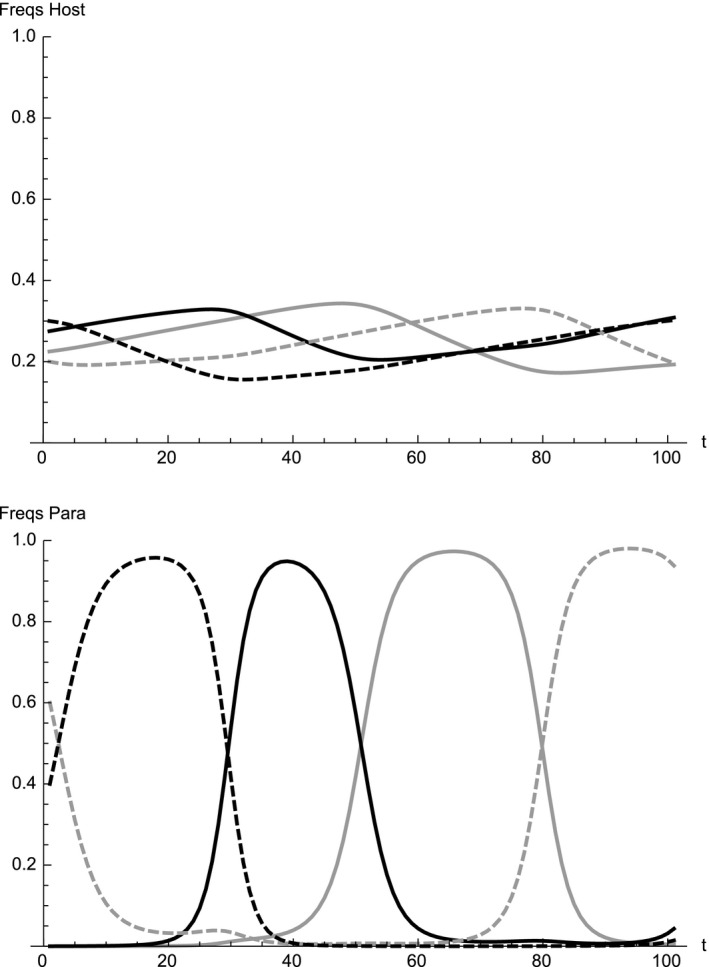
Spread of immaculate white and blue egg in the parasite population when mutation occurs in spottiness, starting from an initial state that immaculate white and blue egg are absent in the parasite population. Initial frequencies are set arbitrarily as (*h*_10_, *h*_20_, *h*_30_, *h*_40_) = (0.225, 0.275, 0.2, 0.3) and (*p*_10_, *p*_20_, *p*_30_, *p*_40_) = (0, 0, 0.3, 0.2). Mutation probabilities and other parameters are the same as in Figure 2. Immaculate white, immaculate blue, white with spots and blue with spots is shown in solid gray, solid black, dashed gray and dashed black, respectively

Considering that the rufescent prinia has four egg types and is capable of recognizing and rejecting unlike eggs in terms in both color and spottiness, cuckoo females producing immaculate white or blue eggs are expected to increase in frequency because they can successfully utilize the prinia nests. Figure 4 shows the increase in frequency of parasites with immaculate white and blue eggs in the presence of the spottiness mutation, starting from an initial state where immaculate white and blue eggs are absent in the parasite population. Immaculate parasite eggs are produced by mutation and they steadily increase in frequency and eventually oscillate around the equilibrium (3) or converge to it depending on the size of the mutation probabilities (see Figure 2). Therefore, if spottiness mutation can occur in the plaintive cuckoo, cuckoos with immaculate white or blue eggs will increase in frequency.

## 4 DISCUSSION

Clear polymorphism in egg phenotype observed in the rufescent prinia and the plaintive cuckoo prompts us to question how these polymorphisms are maintained. We built a population genetic model for the four egg types with a two‐dimensional trait including color (white or blue) and spottiness (immaculate or spots) in order to answer this question. The model analysis shows the possibility that the frequency dynamics exhibit oscillation around the equilibrium where all the types are present with equal frequency and that mutation can stabilize the equilibrium. Although Liang et al. (2012) did not consider egg phenotype mutation, we reached similar conclusions to those of their study, except that we also found that mutation can stabilize the frequency dynamics.

Immaculate white and blue eggs were not found in the observed plaintive cuckoos (Yang, Huang, et al., 2016) and these phenotypes cannot be maintained at equilibrium in the presence of the four egg types of the rufescent prinia (Figure 3). However, no quantitative data are yet available to suggest any trend in the frequency change of the four egg types in the rufescent prinia population. Monitoring the prinia–cuckoo interaction over a long time scale would be worthwhile research to test such a possibility.

Apparent absence of immaculate white and blue eggs in the plaintive cuckoo population may be explained by very low frequencies that prevented detection in field samples given our small sample size (Yang, Huang, et al., 2016). The model analysis has shown that the oscillation period is roughly proportional to the inverse of the square root of the parasitism rate *P*. If *P* is low, as in the case of the common cuckoo parasitism on parrotbills (4.3%, *n* = 555; Yang et al., 2010), the period could be several hundred generations (about 100 generations in Figure 2 where *p* = 0.05). We suggest that immaculate eggs might eventually appear and increase in frequency in plaintive cuckoo populations. Again, long‐term monitoring is needed to confirm whether immaculate eggs are really absent in the plaintive cuckoo population, or if they are present, but at a very low frequency.

Our model assumes closed populations where no gene flow from outside occurs. However, the plaintive cuckoo may have immaculate eggs in local areas where no observations have been made and a geographic frequency cline of immaculate and spotted eggs may exist as has been shown in a parrotbill species (Lee & Jabłoński, 2012). A study of the spatial distribution of the four egg types over a wide geographic scale and an analysis of a model that explicitly considers spatial distribution is needed to more fully understand plaintive cuckoo egg phenotype frequencies.

Discerning the genetic basis of egg phenotype expression is vital in ultimately understanding how egg phenotype polymorphism is maintained. In the common cuckoo (*Cuculus canorus*), egg blueness is inherited asexually in female offspring from the mother (Fossøy et al., 2016). To date, no genetic study has been done on the rufescent prinia and the plaintive cuckoo. Furthermore, no estimate is available for the color change (white or blue) or spottiness (absence or presence) mutation probabilities. In this paper, we have simply used an estimate of per‐locus mutation rates on an order ranging from 10^−4^ to 10^−6^ (Bürger et al., 2006). Avian brood parasitism can be an ideal system because both the host and the parasite have life spans of similar length and hence evolutionary changes in egg phenotype may pace in parallel (Liang et al., 2012).

Two *Prinia* species exist that are closely related to the rufescent prinia in South China: the plain prinia (*P. inornata*) and the gray‐breasted prinia (*P. hodgsonii*). The plain prinia lays white or blue eggs with reddish spots (Wang et al., 2016), seemingly a subset of the four egg types observed in the rufescent prinia. Egg phenotype of the gray‐breasted prinia is unknown. Further comparative study to describe egg phenotype of these two and other closely related species would shed light on the genetic system of egg phenotype expression.

The plaintive cuckoo parasitizes the common tailorbird, a species that shows clear dimorphism with white and blue eggs with reddish spots (Yang, Huang et al. 2016). It could be that an apparent absence of the two egg types in the plaintive cuckoo may have resulted from a parasitic adaptation specialized for the common tailorbird. However, it remains unknown whether unique races of the plaintive cuckoo population exist, each of which is specialized on a particular host species. Empirical and theoretical studies that focus on such races are needed.

In the presence of egg polymorphism, the manner of parasitism can be a crucial determinant in the successful reproduction of the parasite. In order to ensure egg acceptance, cuckoo females should parasitize only host nests where egg phenotype matches. Although this “phenotype matching” parasitic behavior is intuitively appealing, previous empirical studies have shown conflicting results (Antonov et al., 2012; Cherry et al., 2007; Honza et al., 2014; Liang et al., 2016; Yang et al., 2015; Yang, Huang et al., 2016); Yang, Wang, Liang, Møller, 2016). In this model, we have assumed that parasites choose host nests randomly, irrespective of egg phenotype. However, nonrandom parasitism based on phenotype matching could critically affect frequency dynamics. Further study that explicitly considers nonrandom parasitism is needed.

Distinct polymorphism in egg phenotype may be a more universal phenomenon than previously expected in avian brood parasitism (Kim et al., 1995; Lee & Jabłoński, 2012; Lee & Yoo, 2004; Lee et al., 2005; Yang, Li, Liang, Møller 2016; Yang, Huang et al. 2016; Yang et al., 2010). Further study is needed, focusing on genetics and long‐term monitoring, in order to fully understand how polymorphism has evolved and is maintained in avian brood parasitism.

## CONFLICT OF INTEREST

The authors have declared that no competing interests exist.

## APPENDIX 1

### DERIVATION OF THE MODEL

Hosts with egg type *i* can reproduce successfully if they are not parasitized with probability 1−*P*, or if they are parasitized, for example, by a parasite *j* with probability *P p*_*j*_ *,* but successfully reject the parasitism with probability 1−*A*(*i*,* j*). Thus, fitness of the host type *i*,* w*_H*i*_, is given as

$$w_{Hi}=\left(1-P\right)+P\sum_{j=1}^{4}p_{j}\left\{1-A(i,\;\;j)\right\}$$

where the second term of the right‐hand side is summed over all possible parasite types.

Parasites with egg type *j* can reproduce only if the host accepts their eggs. Thus, fitness of the parasite type *j*,* w*_P*j*_, is given as

$$w_{Pj}=P\sum_{i=1}^{4}h_{i}A\left(i,\;\;j\right)$$

the sum of possible target host types.

The average fitness of the host and the parasite is then given as

$${\bar{w}}_{H}=\sum_{i=1}^{4}h_{i}w_{Hi}\;\text{and}\;{\bar{w}}_{P}=\sum_{j=1}^{4}p_{j}w_{Pj},$$

respectively.

Frequencies after host recognition against unlike eggs, **h** ^s^ and **p** ^s^, are given as follows:

$${\text{h}}^{s}=\frac{1}{{\bar{w}}_{h}}W_{H}\text{h}\;\text{and}\;{\text{p}}^{s}=\frac{1}{{\bar{w}}_{P}}W_{p}\text{p}$$

in matrix and vector notation where **W**_H_ and **W**_P_ are the selection matrices of the host and the parasite, respectively, which are given as follows:

$$\begin{array}{c} {\text{W}}_{H}=\left(\begin{array}{cccc} w_{H1} & 0 & 0 & 0\\ 0 & w_{H2} & 0 & 0\\ 0 & 0 & w_{H3} & 0\\ 0 & 0 & 0 & w_{H4} \end{array}\right)\text{and}\;\;{\text{W}}_{P}=\left(\begin{array}{cccc} w_{P1} & 0 & 0 & 0\\ 0 & w_{P2} & 0 & 0\\ 0 & 0 & w_{P3} & 0\\ 0 & 0 & 0 & w_{P4} \end{array}\right) \end{array}$$

Mutations of the color (white or blue) and the spottiness (absence or presence) can be represented by a transition matrix **M** whose (*i*,* j*) element denotes the probability that a type *j* mutates to type *i* (*i*,* j* = 1, 2, 3, 4).

$$\begin{array}{c} \text{M}=\left(\begin{array}{cccc} 1-m_{c}-m_{s} & m_{c} & m_{s} & 0\\ m_{c} & 1-m_{c}-m_{s} & 0 & m_{s}\\ m_{s} & 0 & 1-m_{c}-m_{s} & m_{c}\\ 0 & m_{s} & m_{c} & 1-m_{c}-m_{s} \end{array}\right) \end{array}$$

where double mutations in both color and spottiness are assumed to be negligible.

Frequencies after mutation are then given as **M h** ^s^ and **M p** ^s^ using the mutation matrix for the host and the parasite, respectively. This results in equations (1) and (2).

### POSSIBLE EQUILIBRIA

Without mutations (*m*_c_ = *m*_s_ = 0), equilibria **h*** and **p*** can be derived by solving the following equations:

$${\text{h}}^{*}=\frac{1}{{\bar{w}}_{H}}W_{H}h^{*}\;\text{and}\;{\text{p}}^{*}=\frac{1}{{\bar{w}}_{P}}W_{P}p^{*}.$$

For h* and p* to be in equilibrium of (1) and (2) when mutations occur (*m*_c_, *m*_s _> 0), the following equations have to be satisfied:

$${\text{h}}^{*}=\text{M}\;\;\;{\text{h}}^{*}\;\text{and}\;{\text{p}}^{*}=\text{M}\;\;\;{\text{p}}^{*}$$

Solving these equations results in **h*** and **p*** having equal frequency for all four types.

### LOCAL STABILITY ANALYSI*S*

Linearizing the dynamics (1) and (2) around the equilibrium (3) results in a community matrix that has two zero and six complex eigenvalues.

$$\lambda_{1}=\lambda_{2}=0$$

$$\lambda_{3,4}=\left(1-2m_{c}\right)\left\{1\pm i(A_{0}-A_{c}+A_{s}-A_{\mathrm{cs}})\beta \right\}$$

$$\lambda_{5,6}=\left(1-2m_{s}\right)\left\{1\pm i(A_{0}+A_{c}-A_{s}-A_{\mathrm{cs}})\beta \right\}$$

$$\lambda_{7,8}=\left(1-2m_{c}-2m_{s}\right)\left\{1\pm i(A_{0}-A_{c}-A_{s}+A_{cs})\beta \right\}$$

where

$$\beta =\sqrt{\frac{P}{\left(A_{0}+A_{c}+A_{s}+A_{\mathrm{cs}}\right)\left\{4-P\left(A_{0}+A_{c}+A_{s}+A_{\mathrm{cs}}\right)\right\}}}$$

is a positive real value. The absolute value of the six complex eigenvalues can be less than unity when mutation probabilities *m*_c_ and *m*_s_ are larger than thresholds *m*_c_* and *m*_s_*, respectively. These threshold values can be obtained as maximum solutions *m*_c_, *m*_s_ of |λ_3,4_| = 1, |λ_5,6_| = 1, and |λ_7,8_| = 1. When *m*_c_ and *m*_s_ are less than thresholds *m*_c_* and *m*_s_*, respectively, all of the six complex eigenvalues are less than unity in absolute value and the equilibrium (3) is locally stable. Otherwise, it is unstable. The imaginary part ω of these complex eigenvalues determines the oscillation period *T* when perturbed around the equilibrium as *T* = 2π/ω.

## References

[ece33090-bib-0001] Antonov, A. , Stokke, B. G. , Fossøy, F. , Ranke, P. S. , Liang, W. , Yang, C. , … Røskaft, E. (2012). Are cuckoos maximizing egg mimicry by selecting host individuals with better matching egg phenotypes? PLoS ONE, 7, e31704 https://doi.org/(10.1371/journal.pone.0031704). 22384060 10.1371/journal.pone.0031704 PMC3285637

[ece33090-bib-0002] Brooke, M. D. , & Davies, N. B. (1988). Egg mimicry by cuckoos *Cuculus canorus* in relation to discrimination by hosts. Nature, 335, 630–632. https://doi.org/10.1038/335630a0.

[ece33090-bib-0003] Bürger, R. , Willensdorfer, M. , & Nowak, M. A. (2006). Why are phenotypic mutation rates much higher than genotypic mutation rates? Genetics, 172, 197–206. 16143614 10.1534/genetics.105.046599 PMC1456147

[ece33090-bib-0004] Cherry, M. I. , Bennett, A. T. D. , & Moskát, C. (2007). Do cuckoos choose nests of great reed warblers on the basis of host egg appearance? Journal of Evolutionary Biology, 20, 1218–1222. https://doi.org/10.1111/j.1420-9101.2007.01308.x. 17465931 10.1111/j.1420-9101.2007.01308.x

[ece33090-bib-0005] Davies, N. B. (2000). Cuckoos, cowbirds and other cheats. London, UK: T & AD Poyser.

[ece33090-bib-0006] Davies, N. B. (2011). Cuckoo adaptations: Trickery and tuning. Journal of Zoology, 284, 1–14. https://doi.org/10.1111/j.1469-7998.2011.00810.x.

[ece33090-bib-0007] Davies, N. B. , & Brooke, M. L. (1988). Cuckoos versus reed warblers. Adaptations and counter‐adaptations. Animal Behaviour, 36, 262–284.

[ece33090-bib-0008] Fossøy, F. , Sorenson, M. D. , Liang, W. , Ekrem, T. , Moksnes, A. , Møller, A. P. , … Stokke, B. G. (2016). Ancient origin and maternal inheritance of blue cuckoo eggs. Nature Communications, 7, 10272 https://doi.org/10.1038/ncomms10272. 10.1038/ncomms10272 PMC4729921 26754355

[ece33090-bib-0009] Gibbs, H. L. , Sorenson, M. D. , Marchetti, K. , Brooke, M. L. , Davies, N. B. , & Nakamura, H. (2000). Genetic evidence for female host‐specific races of the common cuckoo. Nature, 407, 183–186. 11001055 10.1038/35025058

[ece33090-bib-0010] Gosler, A. G. , Barnett, P. R. , & Reynolds, S. J. (2000). Inheritance and variation in eggshell patterning in the great tit *Parus major* . Proceedings. Biological Sciences, 267, 2469–2473. 11197121 10.1098/rspb.2000.1307 PMC1690839

[ece33090-bib-0011] Higuchi, H. (1998). Host use and egg color of Japanese cuckoos In Rothstein S. I., & Robinson S. K. (Eds.), Parasitic Birds and Their Hosts: Studies in Coevolution (pp. 80–93). Oxford, UK: Oxford University Press.

[ece33090-bib-0012] Honza, M. , Moksnes, A. , Røskaft, E. , & Stokke, B. G. (2001). How are different common cuckoo *Cuculus canorus* egg morphs maintained? An evaluation of different hypotheses. Ardea, 89, 341–352.

[ece33090-bib-0013] Honza, M. , Sulc, M. , Jelínek, V. , Pozgayová, M. , & Procházka, P. (2014). Brood parasites lay eggs matching the appearance of host clutches. Proceedings of the Royal Society B, 281(1774), 20132665. 24258721 10.1098/rspb.2013.2665 PMC3843844

[ece33090-bib-0014] Kim, C. H. , Yamagishi, S. , & Won, P. O. (1995). Egg‐color dimorphism and breeding success in the crow tit (*Paradoxornis webbiana*). Auk, 112, 831–839.

[ece33090-bib-0015] Lee, J. W. , & Jabłoński, P. G. (2012). Egg color polymorphism and morph‐ratio variation in Korean populations of the vinous‐throated parrotbill. Chinese Birds, 3, 312–319.

[ece33090-bib-0016] Lee, J. W. , Kim, D. W. , & Yoo, J. C. (2005). Egg rejection by both male and female vinous‐throated parrotbills *Paradoxornis webbianus* . Integrative Biosciences, 9, 211–213.

[ece33090-bib-0017] Lee, J. W. , & Yoo, J. C. (2004). Effect of host egg color dimorphism on interactions between the vinous‐throated parrotbill (*Paradoxornis webbianus*) and common cuckoo (*Cuculus canorus*). Korean Journal of Biological Sciences, 8, 77–80.

[ece33090-bib-0018] Liang, W. , Yang, C. , Stokke, B. G. , Antonov, A. , Fossøy, F. , Vikan, J. R. , … Takasu, F. (2012). Modelling the maintenance of egg polymorphism in avian brood parasites and their hosts. Journal of Evolutionary Biology, 25, 916–929. https://doi.org/10.1111/j.1420-9101.2012.02484.x. 22404333 10.1111/j.1420-9101.2012.02484.x

[ece33090-bib-0019] Liang, W. , Yang, C. C. , & Takasu, F. (2016). Modeling the cuckoo's brood parasitic behavior in the presence of egg polymorphism. Journal of Ethology, 34(2), 127–132. https://doi.org/10.1007/s10164-015-0455-3.

[ece33090-bib-0020] Moksnes, A. , & Røskaft, E. (1995). Egg‐morphs and host preference in the common cuckoo (*Cuculus canorus*): An analysis of cuckoo and host eggs from European museum collections. Journal of Zoology, 236, 625–648. https://doi.org/10.1111/j.1469-7998.1995.tb02736.x.

[ece33090-bib-0021] Moksnes, A. , Røskaft, E. , Braa, A. T. , Korsnes, L. , Lampe, H. M. , & Pedersen, H. C. (1991). Behavioural responses of potential hosts towards artificial cuckoo eggs and dummies. Behaviour, 116, 64–89.

[ece33090-bib-0022] Moskát, C. , & Honza, M. (2002). European Cuckoo *Cuculus canorus* parasitism and host's rejection behaviour in a heavily parasitized great reed warbler *Acrocephalus arundinaceus* population. Ibis, 144, 614–622.

[ece33090-bib-0023] Murray, J. D. (2007). Mathematical biology: I. An introduction, 3rd ed. Berlin: Springer.

[ece33090-bib-0024] Øien, I. J. , Moksnes, A. , & Røskaft, E. (1995). Evolution of variation in egg color and marking pattern in European passerines: Adaptations in a coevolutionary arms race with the Cuckoo *Cuculus canorus* . Behavioral Ecology, 6, 166–174.

[ece33090-bib-0025] Rothstein, S. I. (1975). Evolutionary rates and host defenses against avian brood parasitism. American Naturalist, 109, 161–176.

[ece33090-bib-0026] Rothstein, S. I. (1990). A model system for co‐evolution: Avian brood parasitism. Annual Review of Ecology and Systematics, 21, 481–508.

[ece33090-bib-0027] Seger, J. (1988). Dynamics of some simple host‐parasite models with more than two genotypes in each species. Philosophical Transaction of the Royal Society B: Biological Sciences, 319, 541–555. 10.1098/rstb.1988.0064 2905491

[ece33090-bib-0028] Soler, M. (2014). Long‐term coevolution between avian brood parasites and their hosts. Biological Reviews, 89, 688–704. 24330159 10.1111/brv.12075

[ece33090-bib-0029] Stokke, B. G. , Moksnes, A. , & Røskaft, E. (2002). Obligate brood parasites as selective agents for evolution of egg appearance in passerine birds. Evolution, 56, 199–205. 11913664 10.1111/j.0014-3820.2002.tb00861.x

[ece33090-bib-0030] Stokke, B. G. , Takasu, F. , Moksnes, A. , & Røskaft, E. (2007). The importance of clutch characteristics and learning for antiparasite adaptations in hosts of avian brood parasites. Evolution, 61, 2212–2228. 17767591 10.1111/j.1558-5646.2007.00176.x

[ece33090-bib-0031] Takasu, F. (2003). Co‐evolutionary dynamics of egg appearance in avian brood parasitism. Evolutionary Ecology Research, 5, 345–362.

[ece33090-bib-0032] Takasu, F. , & Moskát, C. (2011). Modeling the consequence of increased host tolerance toward avian brood parasitism. Population Ecology, 53, 187–192.

[ece33090-bib-0033] Tanaka, D. K. (2016). Polymorphism in avian brood parasitism: A coevolutionary perspective. Ornithological Science, 15, 133–140.

[ece33090-bib-0034] Wang, L. , Liang, W. , Yang, C. , Cheng, S. , Hsu, Y. , & Lu, X. (2016). Egg rejection and clutch phenotype variation in the plain prinia Prinia inornata. Journal of Avian Biology, 47, 788–794.

[ece33090-bib-0035] Yang, C. , Huang, Q. , Wang, L. , Jiang, A. , Stokke, B. G. , Fossøy, F. , … Møller, A. P. (2016). Plaintive cuckoos do not select tailorbird hosts that match the phenotypes of their own eggs. Behavioral Ecology, 27(3), 835–841. https://doi.org/10.1093/beheco/arv226.

[ece33090-bib-0036] Yang, C. , Li, Z. , Liang, W. , & Møller, A. P. (2016). Egg polymorphism and egg discrimination in the Daurian redstart (*Phoenicurus auroras*), a host of the common cuckoo (*Cuculus canorus*). Ornithological Science, 15, 127–132.

[ece33090-bib-0037] Yang, C. , Liang, W. , Cai, Y. , Shi, S. , Takasu, F. , Møller, A. P. , … Stokke, B. G. (2010). Coevolution in action: Disruptive selection on egg colour in an avian brood parasite and its host. PLoS ONE, 5, e10816. 20520815 10.1371/journal.pone.0010816 PMC2877083

[ece33090-bib-0038] Yang, C. , Takasu, F. , Liang, W. , & Møller, A. P. (2015). Why cuckoos should parasitize parrotbills by laying eggs randomly rather than laying eggs matching the egg appearance of parrotbill hosts? Avian Research, 6, 5 https://doi.org/10.1186/s40657-015-0014-1.

[ece33090-bib-0039] Yang, C. , Wang, L. , Liang, W. , & Møller, A. P. (2016). Do common cuckoos (*Cuculus canorus*) possess an optimal laying behavior to match their own egg phenotype to that of their oriental reed warbler (*Acrocephalus orientalis*) hosts? Biological Journal of the Linnean Society, 117, 422–427.

